# Patients unfit for neoadjuvant therapy may still undergo resection of locally advanced esophageal or esophagogastric junctional cancer with acceptable oncological results

**DOI:** 10.1097/IJ9.0000000000000009

**Published:** 2017-01-13

**Authors:** J. Robert O’Neill, Ewan D. Kennedy, Vicki Save, Barbara Langdale-Brown, Lucy Wall, Richard J.E. Skipworth, Simon Paterson-Brown

**Affiliations:** Departments of a General Surgery; b Pathology, Royal Infirmary of Edinburgh; c Department of Oncology, Western General Hospital, Edinburgh, UK

**Keywords:** Esophageal cancer, Esophagogastric junctional cancer, Neoadjuvant chemotherapy, Mandard

## Abstract

Neoadjuvant chemotherapy (NA) is routinely offered to patients undergoing resection for locally advanced (≥cT3Nx or cTxN+) esophageal or esophagogastric junctional (EGJ) cancer in the United Kingdom. Patients with comorbidity precluding the use of NA can be considered for resection yet the effect of omitting NA on survival is unclear.

**Methods:**

Retrospective review of prospectively collected clinical data from patients undergoing attempted curative therapy for ≥cT3Nx or cTxN+ esophageal or EGJ (Siewert type I-III) cancer between 2001 and 2013.

**Results:**

NA was commenced in 289 patients and primarily comprised 2 cycles of cisplatin and 5-fluorouracil (264 patients, 91%). Surgery alone was planned for 82 patients with NA omitted due to comorbidity. Patients undergoing surgery alone were matched for clinical variables and stage with those undergoing NA but were significantly older (mean=8 y, *P*<0.001). NA was associated with an improved median overall survival of 28.7 months, compared with 20.9 months for patients undergoing surgery alone (*P*=0.008). Patients undergoing surgery alone had a 90-day postoperative mortality rate of 10% compared with 3% for those undergoing NA (*P*=0.011). In patients discharged postoperatively, the median overall survival benefit of NA was 2.7 months (*P*=0.048). Those 19% of patients experiencing a significant histologic response to NA demonstrated further improved survival.

**Conclusions:**

NA improves survival in patients undergoing resection for locally advanced esophageal or EGJ cancer; however, the median benefit is <3 months in patients discharged postoperatively. Patients precluded from NA achieve acceptable oncological results but experience a higher risk of perioperative mortality.

## Abstract 2

Supplemental Digital Content is available in the text.

## Introduction

Esophageal cancer is the eighth most common cancer and sixth leading cause of cancer death worldwide 1. Radical surgical resection remains an essential component of curative therapy yet even with a microscopically complete (R0) resection, many patients relapse with systemic metastases and <40% survive 5 years from diagnosis 2.

Neoadjuvant chemotherapy (NA) has been proposed to improve survival due to tumor downstaging 3–5, and treatment of systemic micrometastases 6. A meta-analysis reported an absolute 2-year survival advantage of 5.1% associated with the use of NA compared with surgery alone for esophageal cancer 7. In the United Kingdom, NA is therefore considered the standard of care for patients undergoing resection for locally advanced (≥clinical T3; ≥cT3 and/or node-positive; cN+) esophageal or esophagogastric junctional (EGJ) cancer 8. A significant survival benefit has also been identified after neoadjuvant chemoradiotherapy and the optimal preoperative regime is the topic of ongoing research 9–11.

The neoadjuvant use of cisplatin and 5-fluorouracil (5-FU) confers a significant risk of cardiac, renal, and other toxicities, which may impair the ability to withstand subsequent major surgery 12,13, and NA treatment-related mortalities are reported 14. The response to NA is variable and evidence is mounting that the majority of the survival benefit from NA is derived by a subset of patients exhibiting a significant histologic response 3,15–21. The remaining patients experience little or no benefit, yet still the potential harm from NA toxicity and delay to surgical resection 22,23. Positron emission tomography (PET) has been used to assess early tumor response during NA chemotherapy, although the accuracy of this technique has yet to be determined and unfortunately, no test has been approved to predict if patients with esophageal or EGJ cancer will respond to NA 24–27.

Histopathologic examination of the final resected specimen remains the standard method of determining tumor regression 15,28. Several systems using a tiered tumor regression score have been proposed and although none have gained universal acceptance, the most widely used method is that of Mandard et al^28^ (Supplemental Table 1, Supplemental Digital Content 1, http://links.lww.com/IJSO/A0) 29,30.

Contemporary improvements in perioperative care allow patients with locally advanced esophageal cancer yet comorbidities precluding the use of NA, to undergo esophageal resection with acceptable in-hospital mortality rates 31. The oncological outcome for these patients after surgery alone is not clear, as data from historical cohorts before the routine use of NA may be confounded by poorer quality preoperative staging and perioperative care.

The aim of this single center, retrospective cohort study was to determine the contemporary outcome after surgery alone for patients with locally advanced esophageal cancer (≥cT3Nx or cTxN+) precluded from NA due to comorbidity and compare it with clinically stage-matched patients undergoing NA and surgery.

## Methods

Deidentified treatment and outcome data were retrospectively obtained from a prospectively maintained audit database including patients undergoing attempted curative therapy for mid, distal esophageal or EGJ (Siewert type I-III) adenocarcinoma, or squamous cell carcinoma between January 2001 and December 2013 at a single tertiary referral center. Additional patient consent and ethical approval were not required as only data obtained as part of routine clinical care were used for this study. As standards of clinical and pathologic staging changed during the study period, original staging investigations, pathology reports and, if necessary, specimens were re-examined and reported using the criteria of the 7th Edition of the TNM staging manual 32.

All patients considered fit for surgical resection were staged by a multidisciplinary team comprising esophagogastric surgeons, gastroenterologists, oncologists, radiologists, and pathologists using a combination of endoscopy, chest and abdominal computed tomography (CT), CT-PET, endoscopic ultrasound (EUS), and laparoscopy. The clinical stage and tumor length were derived from the EUS, or if no EUS was conducted, from a combination of CT, CT-PET, and laparoscopy, using the highest cT and cN stage.

Patients staged as cT1N0 or cT2N0 were not considered for NA and were excluded from analysis. Those patients staged as cT3Nx, cT4aNx, or cTxN+ with resectable disease and no contraindications were offered NA followed by surgery.

Patients with esophageal or Siewert type I or II EGJ tumors were given NA comprising 2 cycles of cisplatin (80 mg/m^2^) on day 1 and 5-FU (1 g/m^2^) on days 1 to 4 every 21 days (2×CF). Resection then comprised an en bloc esophageal and gastric dissection with a mediastinal and upper abdominal lymphadenectomy (Ivor-Lewis esophagogastrectomy) or, for occasional bulky type II tumors, a lower mediastinal and upper abdominal lymphadenectomy was performed with a left thoracoabdominal esophagogastrectomy. Rarely, for frail patients, a transhiatal approach was used.

Patients with Siewert type III EGJ tumors were given epirubicin 50 mg/m^2^ on day 1, cisplatin 60 mg/m^2^ on day 1, and capecitabine 1250 mg/m^2^/d continuously (ECX) for 3, 21-day preoperative cycles. Resection then comprised a left thoracoabdominal esophagogastrectomy, or extended total gastrectomy.

A small proportion of patients with esophageal and Siewert type I and II EGJ tumors received 4 cycles of preoperative ECX as part of a clinical trial. Neoadjuvant radiotherapy was not used. All resections were tailored to the tumor location and patient comorbidity and were planned for between 4 and 8 weeks after completion of NA.

NA was considered contraindicated in patients with an estimated glomerular filtration rate <60 mL/min, symptomatic ischemic heart disease or peripheral vascular disease, a history of myocardial infarction or cerebrovascular accident within the previous 12 months, previous peripheral vascular disease requiring surgery or significant hearing impairment already requiring a hearing aid. Additional patients with cardiovascular comorbidities less significant than those above were reviewed in an oncology clinic and a collaborative decision reached as to proceeding with NA or going directly to surgery.

A microscopically incomplete (R1) resection was defined as tumor cells within 1 mm of any resection margin, tumor differentiation by the most poorly differentiated area, and pathologic size as the maximum in any dimension 33. Histologic response to NA was retrospectively assessed using the Mandard system 28 (Supplemental Table 1, Supplemental Digital Content 1, http://links.lww.com/IJSO/A0) for a random subgroup of patients undergoing NA and surgery by 2 specialist upper-gastrointestinal pathologists, blinded to clinical and outcome data, through review of the diagnostic slides produced at the time of resection.

Postoperative radiotherapy, chemotherapy, or observation alone were offered by the multidisciplinary team after review of the resection pathology, consideration of patient comorbidities, and postoperative recovery. Recurrence data were obtained from follow-up consultations with date of recurrence defined as date of restaging investigation confirming either local (mediastinal or anastomotic) or distant recurrence. Survival was defined as date of diagnostic biopsy to death or last follow-up with primary or tertiary care, censoring on December 31, 2014.

All statistical analysis was performed using “IBM SPSS Statistics” software (Version 21.0.0, SPSS Inc. Chicago, IL). Categorical variables are illustrated in tables and were compared using the χ^2^ test. Continuous variables were summarized by medians or means if appropriate and 95% confidence intervals included in parenthesis unless otherwise stated.

Univariable survival analysis was performed using the Kaplan-Meier method with a log-rank test of significance for categorical covariates and Cox regression for continuous covariates 2. Statistical significance was defined as a *P*<0.05. Covariates significantly associated with survival on univariable analysis were assessed in a Cox proportional hazards model 2.

## Results

Of the 497 identified patients, 126 were excluded for the following reasons; palliative chemotherapy and salvage surgery (n=3), not considered for NA due to early (cT1N0 or cT2N0) stage (n=47), and clinical staging investigations or pathologic specimens not available for review (n=76). The estimated median survival of the remaining 371 patients was 28.1 months with an estimated 5-year survival rate of 29% and a median follow-up for the 148 survivors of 30 months. The cohort clinical characteristics are summarized in **Table 1**.

**Table 1:**
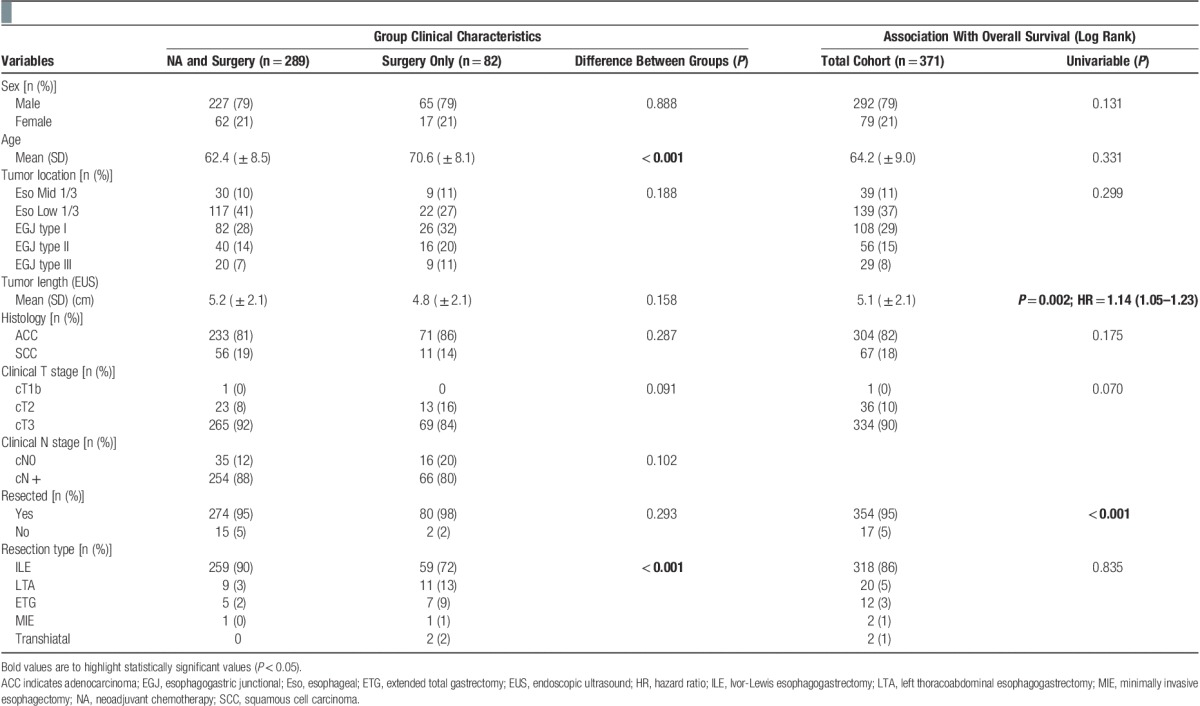
Cohort clinical characteristics and survival analysis.

NA was commenced in 289 patients and 274 proceeded to resection (95%) compared with 98% of the 82 patients planned for surgery alone (*P*=0.293). NA consisted of 2×CF in 264 patients (91%) or 3 cycles (20 patients; 7%) or 4 cycles (5 patients; 2%) of ECX.

Patients going straight to surgery were well matched with those undergoing NA for measured clinical variables and stage but were significantly older (mean difference of 8 y, *P*<0.001). The type of resection, a reflection of tumor position, also differed significantly between groups (*P*<0.001) but neither age nor operative approach were associated with overall survival (**Table 1**). The rate of microscopic (R1) or macroscopic (R2) involvement of resection margins also did not differ significantly between groups (*P*=0.189, **Table 2**) and a mean of 23 nodes were resected in both groups (**Table 2**).

**Table 2:**
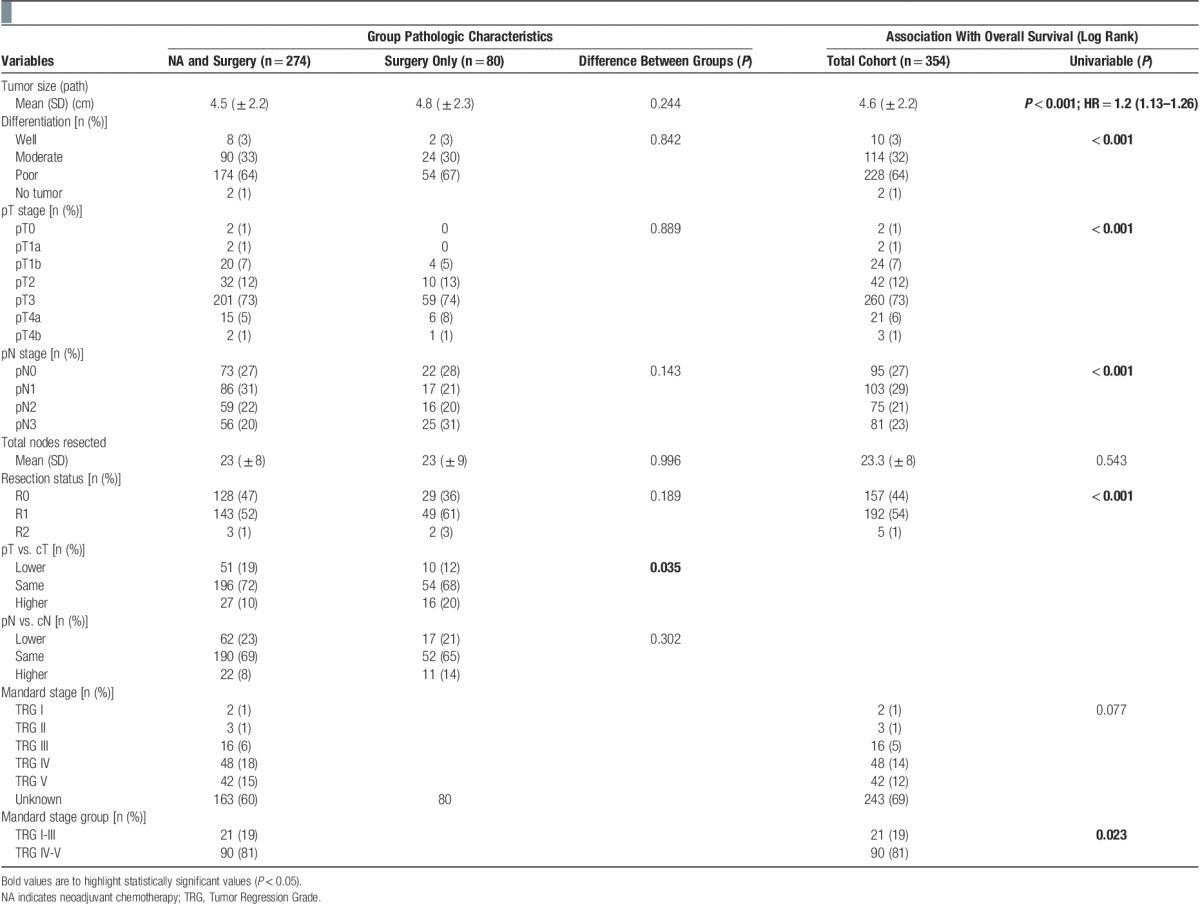
Cohort pathologic characteristics and survival analysis.

**Figure 1:**
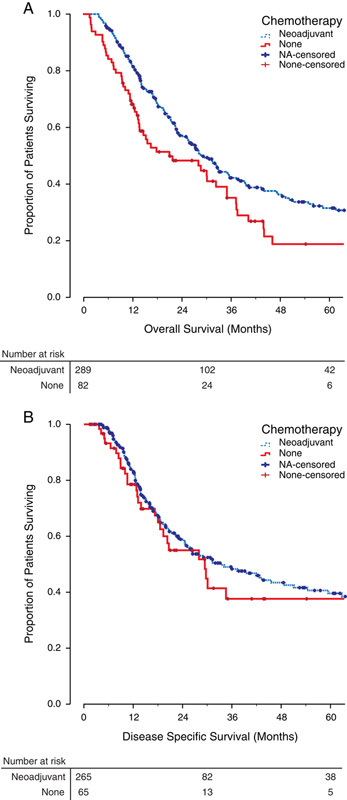
The effect of neoadjuvant chemotherapy on survival. A, Kaplan Meier plot demonstrating overall survival on intention to treat. Estimated 2-year survival rate; neoadjuvant (NA) 57%, none (surgery alone) 48%. Estimated 5-year survival rate; NA 31%, none 19%. B, Kaplan Meier plot demonstrating disease-specific survival on intention to treat. Estimated 2-year survival rate; NA 59%, none 55%. Estimated 5-year survival rate; NA 39%, none 37%.

On intention to treat, NA was associated with a significantly longer median overall survival of 28.7 months (range, 24.3–33.0 mo), compared with 20.9 months (range, 7.2–34.6 mo) for patients undergoing surgery alone (*P*=0.008, **Fig. 1A**). In contrast, disease-specific survival did not differ significantly between groups [NA—median survival of 33.4 mo (range, 22.2–44.5 mo), surgery alone—median survival of 29.4 mo (range, 15.7–43.1 mo); *P*=0.550; **Fig. 1B**].

**Figure 2:**
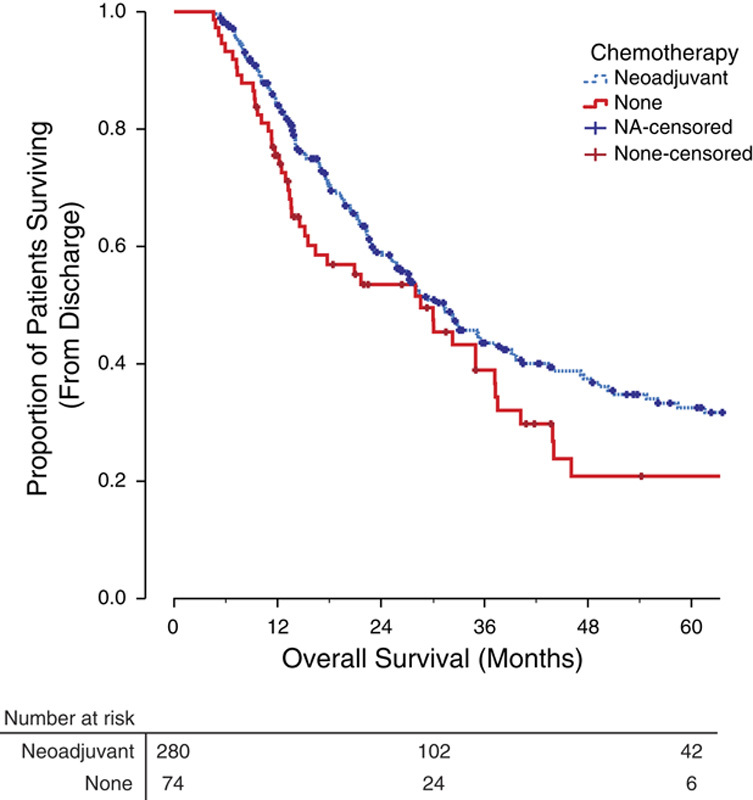
The effect of neoadjuvant chemotherapy on overall survival in patients surviving to postoperative discharge. Kaplan Meier plot demonstrating overall survival. Estimated 2-year survival rate; neoadjuvant (NA) 59%, none 53%. Estimated 5-year survival rate; NA 32%, none 21%.

The overall 90-day postoperative mortality rate was 4.8% (17/354 patients). Patients going straight to surgery had a significantly higher postoperative mortality rate of 10% (8/80 patients) compared with 3% (9/274 patients) for those undergoing NA and surgery (χ^2^, *P*=0.011). If those patients dying within 90 postoperative days were excluded, NA was still associated with significantly longer overall survival (median, 31.3 mo; range, 26.3–36.3 mo) over surgery alone (median, 28.6 mo; range, 16.9–40.2 mo; *P*=0.048; **Fig. 2**). As all deaths within 90 days were due to postoperative complications rather than disease recurrences, disease-specific survival was unchanged.

The use of NA was independently predictive of overall survival (hazard ratio, 0.655; range, 0.469–0.914; *P*=0.013) along with pathologic tumor size, pN stage, and resection margin status (Supplemental Table 2, Supplemental Digital Content 2, http://links.lww.com/IJSO/A1). NA has been proposed to downstage esophageal tumors. When pT stage was compared with cT stage, significantly fewer patients were upstaged and more were downstaged after NA compared with surgery alone although the differences were small (**Table 2**; *P*=0.035). There was also a trend toward smaller tumors in the NA group (**Table 2**) but this difference was not statistically significant. No difference was observed between groups when cN and pN stages were compared (**Table 2**; *P*=0.302).

**Figure 3:**
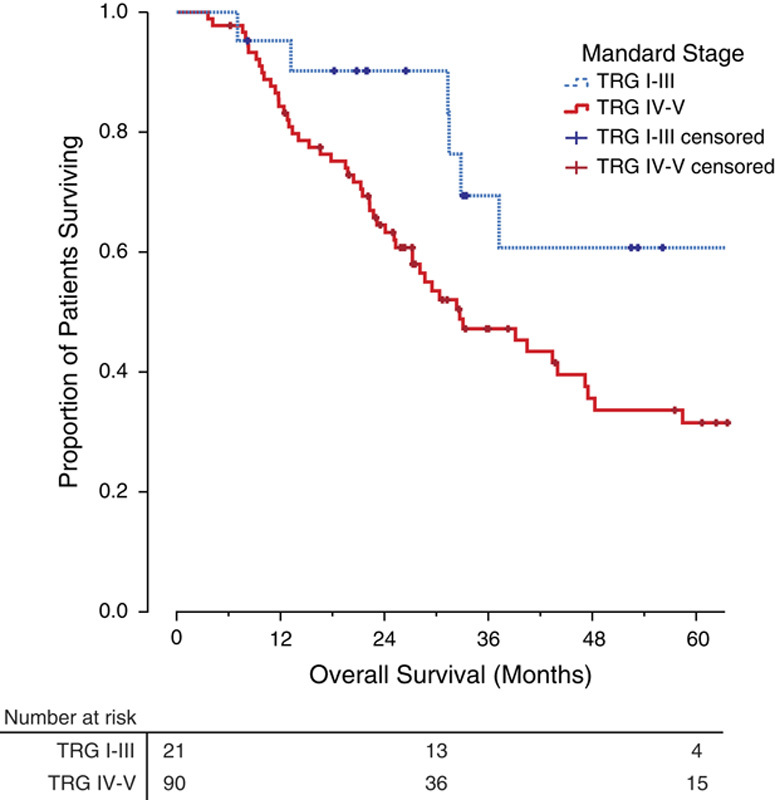
The association of Mandard Tumor Regression Grade (TRG) and overall survival after neoadjuvant chemotherapy and surgery. Kaplan Meier plot demonstrating overall survival. Estimated 2-year overall survival rate; TRG I-III 90%, TRG IV-V 64%. Estimated 5-year overall survival rate; TRG I-III 60%, TRG IV-V 31%.

The histologic response to NA was assessed in a randomly selected subset (40%) of the 274 patients undergoing NA and resection. A significant histologic response (TRG I-III) was observed in 21 patients (19%) and was associated with a significantly improved overall survival (median, 37.2 mo; range, 26.3–48.2 mo) compared with patients exhibiting no significant histologic response (TRG IV-V), [median, 32.7 mo (range, 21.3–44.1 mo); hazard ratio for mortality=0.39 (range, 0.168–0.907); *P*=0.023; **Fig. 3**].

## Discussion

Increasing numbers of patients considered fit enough for surgical resection of ≥cT3Nx or cTxN+ esophageal or EGJ cancer present with comorbidities precluding the use of NA. This retrospective study examined the effect of omitting NA on the survival of these patients.

NA was associated with a median overall survival benefit of 7.8 months (**Fig. 1**). The benefit of NA was reduced to 2.7 months when deaths within 90 postoperative days were excluded (**Fig. 2**). This is likely due to the higher in-hospital mortality rate of 10% in patients undergoing surgery alone compared with 3% in patients undergoing NA before surgery. A similar 90-day, postoperative mortality rate of 11.3% was reported in a study of 212 patients with cT3 or cT4 disease going straight to surgery 34. This likely reflects the greater comorbidity of the group precluded from NA.

No significant difference in disease-specific survival could be determined although a trend in favor of NA was identified. Recurrence data were only available for 89% of the cohort, which reduces the sensitivity to detect a difference. Routine CT scanning was not used during follow-up with investigations instigated on clinical suspicion. Symptomatic recurrences were correspondingly more likely to be identified; however, it is unlikely this introduces a bias in favor of the surgery-alone group. If routine imaging is used during follow-up after esophageal cancer surgery the recorded time to recurrence is still a reflection of the time to diagnostic investigation rather than a precise measure of disease progression.

The finding of fewer patients upstaged and more downstaged in the NA group may be taken as evidence of tumor downstaging (**Table 2**). However, 21% of patients undergoing surgery alone were “downstaged” from cN+ to pN0 echoing published findings from a similar cohort of 82 patients of whom 16% undergoing surgery alone with cN+ disease were classified as pN0 18. A third of patients going straight to surgery in our study were also incorrectly T-staged. This highlights the limited accuracy of current clinical staging methods and caution should be advised before conclusions of downstaging are drawn from comparison of clinical and pathologic stages 35.

The NA group predominantly consisted of patients treated with 2×CF and 19% of a sample of those exhibited a significant histologic response (TRG I-III) with a corresponding longer median overall survival. Although only a sample of those undergoing NA were assessed, our findings are likely to be representative as a similar significant response rate of 15% was reported in 451 patients with esophageal and EGJ cancer undergoing NA with 2×CF and resection as part of the OE05 trial 36. The Radiation Therapy Oncology Group trial 8911 compared surgery alone with preoperative cisplatin and 5-FU and a long-term follow-up study identified no difference in survival. A significant response to chemotherapy was, however, noted in 19% of patients and although this was a radiologic rather than histologic assessment, the responders exhibited similarly significantly improved survival 23.

Several further studies have reported a survival benefit in patients experiencing a significant histologic response to NA. One group reported significant response rates (TRG I-III) of 41% in patients receiving predominantly 3 cycles of ECX and an improved survival in those patients experiencing the best response (TRGI-II) compared with the remainder 18. A similar significant response rate after 4 cycles of ECX was noted in the OE05 trial 36.

This study does have limitations. Although a consecutive series of patients was identified from 1 institution, 76 of the total 450 eligible patients (17%) were excluded due to missing clinical staging investigations, pathology reports, or specimens and this may confound the conclusions through selection bias. Patients undergoing surgery alone were all eligible for chemotherapy on the basis of clinical staging but went straight to surgery due to comorbidity precluding the use of NA. A detailed profile of comorbidity was not prospectively collected but cardiovascular and/or renal disease were the reasons for excluding NA in the majority of cases.

We propose that patient comorbidity is responsible for the higher 90-day postoperative mortality rate in the surgery-alone group. An alternative explanation would be that chemotherapy is protective in the postoperative period. The 47 excluded patients with cT1N0 or cT2N0 disease had a 90-day postoperative mortality rate of 4% (2 patients), however, similar to those undergoing chemotherapy. Similarly, the published trials of NA in esophageal cancer do not support a protective effect on 90-day postoperative mortality 7.

In conclusion, this study reports acceptable oncological outcomes for patients precluded from NA yet undergoing attempted curative surgery for locally advanced esophageal cancer. Therefore, despite higher 90-day postoperative mortality rates, patients with cardiovascular and renal disease should not be denied major esophagogastic cancer resection.

Neoadjuvant therapy was associated with improved survival, although in patients surviving to hospital discharge, this benefit was <3 months. The survival benefit was greater in the 19% of patients exhibiting a significant histologic response to therapy. Pretreatment tests to identify those patients likely to respond are urgently required.

## Supplementary Material

Supplemental Digital Content is available for this article. Direct URL citations appear in the printed text and are provided in the HTML and PDF versions of this article on the journal's Website, www.IJSOncology.com.
